# Evolution of Reproductive Morphology in Leaf Endophytes

**DOI:** 10.1371/journal.pone.0004246

**Published:** 2009-01-22

**Authors:** Zheng Wang, Peter R. Johnston, Zhu L. Yang, Jeffrey P. Townsend

**Affiliations:** 1 Department of Ecology and Evolutionary Biology, Yale University, New Haven, Connecticut, United States of America; 2 Herbarium PDD, Landcare Research, Auckland, New Zealand; 3 Key Laboratory of Biodiversity and Biogeography, Kunming Institute of Botany, Chinese Academy of Sciences, Kunming, Yunnan, China; Centre National de la Recherche Scientifique, France

## Abstract

The endophytic lifestyle has played an important role in the evolution of the morphology of reproductive structures (body) in one of the most problematic groups in fungal classification, the Leotiomycetes (Ascomycota). Mapping fungal morphologies to two groups in the Leiotiomycetes, the Rhytismatales and Hemiphacidiaceae reveals significant divergence in body size, shape and complexity. Mapping ecological roles to these taxa reveals that the groups include endophytic fungi living on leaves and saprobic fungi living on duff or dead wood. Finally, mapping of the morphologies to ecological roles reveals that leaf endophytes produce small, highly reduced fruiting bodies covered with fungal tissue or dead host tissue, while saprobic species produce large and intricate fruiting bodies. Intriguingly, resemblance between asexual conidiomata and sexual ascomata in some leotiomycetes implicates some common developmental pathways for sexual and asexual development in these fungi.

## Introduction

Body size and shape are traits that correlate with diverse aspects of the biology of a species, from genetics, physiology and life history to ecology. For this reason, biologists have long been interested in understanding how body size and shape evolve [1]. However, less attention has been focused on the evolution of body size of the estimated 1.5 million species of fungi, perhaps in part because they are found in varied and complex associations with many other groups of organisms in diverse ecosystems [2]–[3]. Until recently, studies of symbiotic relationships between higher fungi and plants have been largely relegated to underground mycorrhizas and root endophytes, most of which are mushroom-forming basidiomycetes. In general, fungal endophytes are fungi that live inside plants without causing apparent symptoms of infection [4], but some saprobic or pathogenic fungi can have endophytic stages in part of or even during the whole of their life history, under specific environmental conditions. Diverse fungal endophytes have been identified thriving within living leaves from all major lineages of ascomycetes, and they represent a significant proportion of total fungal diversity [4]–[5]. Some leaf endophytes/pathogens produce a noticeable reproductive structure (body) during sexual and asexual reproduction, and the highly specialized body shape and reduced body size of leaf endophytes makes the current classification of ascomycetes challenging [6]–[8]. However, form and function are often correlated through change of ecological role, and the mysterious origins of diverse leaf endophytes may be addressed by studies that relate phylogeny, ecology, and morphology to the evolutionary history of fungi.

Ecological role has often been elucidated by studies of form and function evolution in animals and plants, but less frequently in fungi, due to their diversity and lability of form and unresolved phylogenies [9]. Nowhere across the fungal kingdom has this diversity and lability been more challenging to explain than in the Leotiomycetes, which includes many saprobes, plant pathogens, and endophytes [8], [10]–[11]. Their complex reproductive morphologies (sexual ascomata and asexual conidiomata) are highly variable at all taxonomic ranks. Endophytes and pathogenic endophytes (conditional pathogens) are fairly common in the Leotiomycetes, and they are traditionally classified into two groups, the Rhytismatales and Hemiphacidiaceae (Helotiales). For most species in the Rhytismatales the sexual reproductive structure develops within a dark stroma, and the stroma typically immersed within host tissue and sometimes covered by a clypeus made of fungal hyphae. The Hemiphacidiaceae contains conifer pathogens producing small ascomata beneath the leaf surface, the erumpent ascomata pushing back the covering host tissue when matured. Unlike other orders in the Leotiomycetes, classification of the Rhytismatales and Hemiphacidiaceae had been straightforward due to their unique morphology. However, both groups have experienced many taxonomic changes as increasing amounts of molecular sequence data on relevant taxa have been gathered and analyzed [7]–[8], [12].

To sort out the evolutionary history of various endophytic lineages and thereby the corresponding shifts in body size and shape that accompanied these transitions, we analyzed rDNA data from 77 ascomycetes, including taxonomic representation of the two endophytic groups, the Rhytismatales and Hemiphacidiaceae (Leotiomycetes). Analysis of morphology in the light of this phylogenetic data reveals a relationship between body plan and endophytic lifestyle was evident in these fungi: a highly reduced morphology is an adaptation to survival on living leaves on trees, and the adaptation is evident in the morphology of both sexual and asexual reproductive structures.

## Results

### 1. Phylogenetic analyses (Fig. 1)

**Figure 1:**
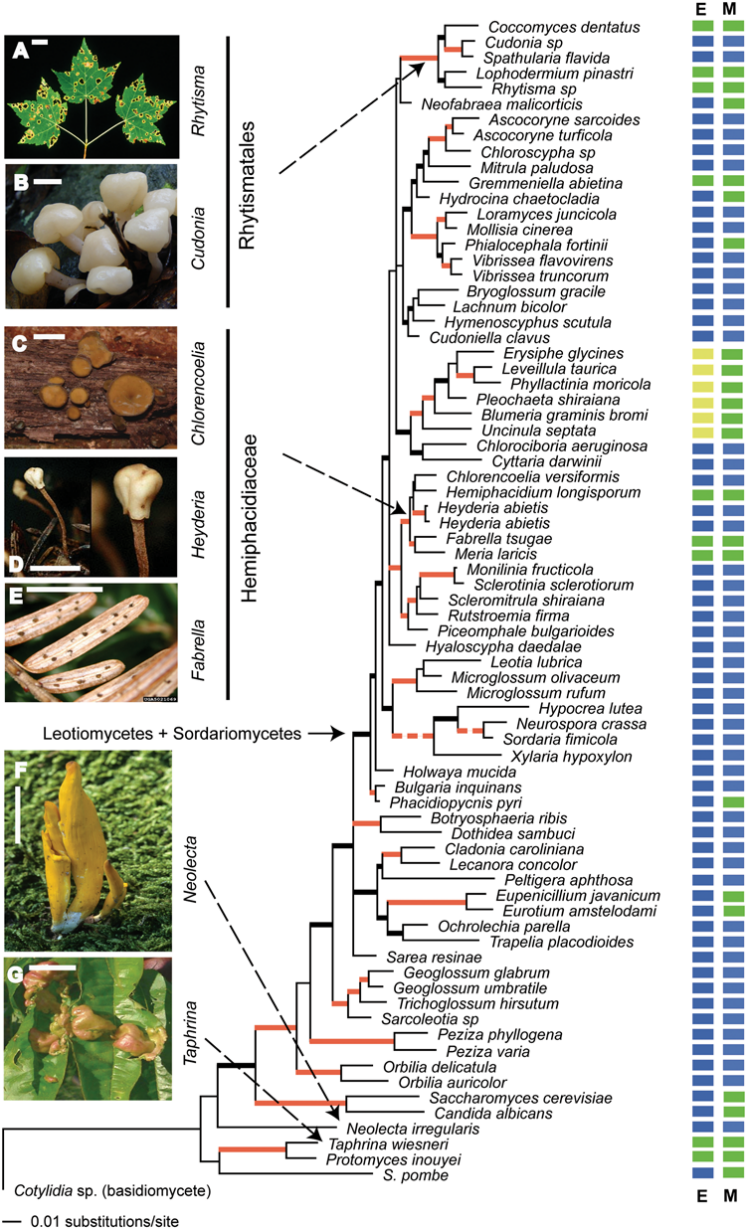
Bayesian 50% majority-rule consensus phylogeny of the Ascomycota based on rDNA. Branches with posterior probabilities ≥0.95 are bold. MP bootstrap values ≥80% are red. Notably long branches are dashed. Column E: Ecological character – leaf endophytic (green), leaf epiphytic (yellowish green), or non-leaf endophytic (blue). Column M: Morphological character – fully developed (blue) or small/reduced (green). Exemplars of ascomata morphology and ecology of fungi are shown (Scale bar = 10 mm): A. Tar-spots of *Rhytisma americanum* on maple leaves (G.W. Hudler/Cornell University). B. *Cudonia* sp. (Z.W. Ge/HKAS). C. *Chlorencoelia versiformis* on fallen wood (Ed Bosman/Connecticut Valley Mycological Society). D. *Heyderia abietis* on fallen needles (J.H. Petersen/MycoKey). E. *Fabrella tsugae* causing needle-blight of hemlock (Pennsylvania Department of Conservation and Natural Resources). F. *Neolecta irregularis* on mossy ground (J. Plischke/NAMA). G. *Taphrina deformans* causing leaf curl on a peach tree (J. Conrad/backyardnature.net).

To infer the phylogeny of leaf endophytes and endophytic pathogens across ascomycetes, parsimony analyses using PAUP 4.0 [13] and Bayesian analyses using MrBayes [14] were performed on the 78 taxa data set, which included 77 representatives of major lineages of ascomycetes and one basidiomycete, *Cotylidia* sp., as the outgroup. Parsimony analyses using the rDNA data set for 78 taxa produced nine equally best trees (length = 4278, CI = 0.349, and RI = 0.509). The clade including the Leotiomycetes and the Sordariomycetes received weak support (56% BP), a result that is consistent with previous studies using diverse molecular markers. Two clades were of interest. The Rhytismatales clade includes saprobic species of *Cudonia* and *Spathularia* and endophytic species of *Coccomyces*, *Rhytisma*, and *Lophodermium*. The Hemiphacidiaceae clade includes saprobic species of *Chlorencoelia* and *Hedydria*, and endophytic species of *Hemiphacidium*, *Fabrella*, and *Meria*. These two clades were strongly supported with bootstrap values of 100% and 97% respectively. Within the Rhytismatales clade, *Cudonia* and *Spathularia* were supported as a clade (100% BP), with *Coccomyces dentatus* as their sister group. Within the Hemiphacidiaceae clade, species of *Fabrella* and *Meria* formed a clade with 86% BP, while *Hemiphacidium longisporum* was located in the clade with *Chlorencoelia versiformis* (57% BP). A majority rule consensus tree of the Bayesian posterior tree set presented almost the same topology as that from parsimony analyses. The Leotiomycetes - Sordariomycetes clade was strongly supported (1.00 PP). The Rhytismatales clade received a support of 1.00 PP, and the Cudonia-Spathularia clade (1.00 PP) was the sister group to *Coccomyces dentatus* (1.00 PP). The Hemiphacidiaceae clade was supported with 1.00 PP as well, and within the clade species of *Heyderia*, *Hemiphacidium*, *Chlorencoelia* formed a clade (1.00 PP) sister to the Fabrella-Meria clade (1.00 PP).

### 2. Analysis of correlation between leaf-living ecology and reduced morphology

After a run of 1 million iterations in BayesTraits [15] to estimate the correlation between leaf-living ecology and reduced morphology, the following harmonic means of the marginal likelihood were obtained, with virtually no difference between runs: ∼71.50 for the model in which leaf-living ecology and reduced morphology are independent, and ∼56.50 for the model in which they are dependent. The log-Bayes Factor is twice the difference of these two harmonic means, equaling ∼30, revealing very strong support (≫5) for the correlation (dependent model) between the leaf-living ecology and reduced fruiting body morphology.

## Discussion

The tar-spot symptoms on leaves of maples and oaks are typical of the highly reduced ascomata of leaf-living species in the Rhytismatales. These develop within a dark stroma immersed within host tissues. In contrast, species of the saprobic family Cudoniaceae produce large, bright ascomata on leaf duff or mossy ground. Except for a similar development style inferred from a reduced membrane covering the young fruiting body of *Cudonia* species, no obvious morphological characters suggest a relationship between the Cudoniaceae and the Rhytismatales [16]. Our results validate previous molecular studies that had suggested that the saprobic family Cudoniaceae lies within the endophytic Rhytismatales [7], [12], [16]. ITS sequences of *Cudonia* were amplified from leaf samples in an investigation of foliar fungal endophytes, but the ITS phylogenies were not resolved for the Cudonia-Rhytismatales clade in that study [5]. Although it is very likely that certain stages in the life cycle of *Cudonia* species are endophytic in leaves of plants, there had been no direct evidence. For the first time, our phylogenetic placement of a bryophilous species of *Cudonia* found recently in Yunnan, China, ecologically links the Cudoniaceae to the endophytic/pathogenic fungi that are so characteristic of the Rhytismatales.

Our data also demonstrate lability of morphology in the Hemiphacidiaceae, another family including both leaf endophytes and pathogens. Previously, all fungi placed in the Hemiphacidiaceae produced small, highly reduced ascomata beneath the surface of leaves. In our analysis, *Heyderia* and *Chlorencoelia* species were placed within this family, despite production by both species of large and fully developed ascomata (Fig. 1).

Similar morphological adaptation is also known within the basal ascomycetes (subphylum Taphrinomycotina). Saprobic *Neolecta* species produce large ascomata on the ground, while pathogenic species of *Taphrina* produce naked asci on the leaf surface [17]. The phylogenetic dependence across all taxa between leaf-living ecology and reduced morphology is strongly significant (Bayes Factor = ∼30). For leaf endophytes and pathogens, a small, covered ascomata is an adaptation to the ecological constraints of limited physical support, strong radiation, and rapid evaporation. For their saprobic relatives, a large and usually stalked fruiting body has been suggested to be advantageous for spore dispersal. This advantage of height and the ensuing spore production pattern may play a role in the evolution of morphology in both ascomycete and basidiomycete (mushroom) forest floor saprobes.

The genetic basis of both sexual and asexual development in diverse organisms has received extensive attention. Many fungi have both asexual and sexual cycles in their life histories. In general, asexually produced conidiomata are very simple and significantly morphologically distinct from the more complex, sexually produced ascomata. But for some leotiomycetes, such as the *Holwaya* and *Ascocoryne*, the conidiomata are prominent and composed of tissues homologous to the ascomata [18]. In contrast, ascomata of endophytic species of the Rhytismatales resemble the simple conidiomata with which they are physically associated [19]. Some of the same genes or pathways may function pleiotropically in the development of both ascomata and conidiomata in these fungi.

One of the central questions in evolutionary developmental biology is how the same genes and often the same pathways may be activated to perform different developmental functions in different tissues and at different times [20]. Simple morphologies without closely-related, significant evolved differences limit the usefulness of model fungi such as yeasts and *Neurospora* in exploring the genetic basis of the development and evolution of intricate morphological structures. Will there be another golden age for fungi in “evo-devo”, after yeasts and *Neurospora*? With many genome projects being completed and new interesting discoveries about the constraints and evolvability of morphology in fungi, it seems possible.

## Materials and Methods

### 1. Taxon sampling

The data matrix contained 77 taxa of the Ascomycota and one basidiomycete *Cotylidia* sp. as the outgroup, constructed from sequences of SSU rDNA, LSU rDNA, and 5.8S rDNA genes (table 1). All sequence data were downloaded from GenBank or were produced by the AFTOL project and used in previous study by Z.W. [8].

**Table 1:** Species examined with information on GenBank accession numbers by DNA locus.

Species	SSU-rDNA	LSU-rDNA	5.8S rDNA
*Ascocoryne sarcoides* (Jacq.) J.W. Groves & D.E. Wilson	AY789387	AJ406399	AY789388
*Ascocoryne turficola* (Boud.) Korf	AY789276	AY789277	AY789278
*Blumeria graminis* (DC.) Speer	AB033476	AB022362	AJ313142
*Botryosphaeria ribis* Grossenb. & Duggar	AF271129	AY004336	AF027744
*Bryoglossum gracile* (P. Karst.) Redhead	AY789419	AY789420	AY789421
*Bulgaria inquinans* (Pers.) Fr.	AY789343	AY789344	AY789345
*Candida albicans* (C.P. Robin) Berkhout	X53497	L28817	AY672930
*Chlorencoelia versiformis* (Pers.) Dixon	AY789350	AY789351	AY789352
*Chlorociboria aeruginosa* (Oeder) C.S. Ramamurthi, Korf & L.R. Batra	AY544713	AY544669	AY755360
*Chloroscypha* sp.	AY544700	AY544656	U92311
*Cladonia caroliniana* (Schwein.) Tuck.	AY584664	AY584640	AF456408
*Coccomyces dentatus* (J. C. Schmidt & Kunze) Sacc.	AY544701	AY544657	N/A
*Cotylidia* sp.	AY705958	AY629317	AY854079
*Cudonia* sp.	AF107343	AF279379	AF433149
*Cudoniella clavus* (Alb. & Schwein.) Dennis	AY789340	AY789341	AY789342
*Cyttaria darwinii* Berk.	U53369	UNPUBL.	UNPUBL.
*Dothidea sambuci* (Pers.) Fr.	AY544722	AY544681	AY883094
*Erysiphe glycines* F. L. Tai	AB120748	AB022397	AB078807
*Eupenicillium javanicum* (J.F.H. Beyma) Stolk & D.B. Scott	U21298	AF263348	U18358
*Eurotium amstelodami* L. Mangin	AB002076	AY213699	AY213648
*Fabrella tsugae* (Farl.) Kirschst.	AF106015	AF356694	U92304
*Geoglossum glabrum* Pers.	AY789316	AY789317	AY789318
*Geoglossum umbratile* Sacc.	AY789302	AY789303	AY789304
*Gremmeniella abietina* (Lagerb.) M. Morelet	AF203456	UNPUBL.	U72259
*Hemiphacidium longisporum* Ziller & A. Funk	UNPUBL.	UNPUBL.	N/A
*Heyderia abietis* (Fr.) Link	AY789288	AY789289	AY789290
*Heyderia abietis*	AY789295	AY789296	AY789297
*Holwaya mucida* (Schulzer) Korf & Abawi	DQ257355	DQ257356	DQ257357
*Hyaloscypha daedaleae* Velen	AY789414	AY789415	AY789416
*Hydrocina chaetocladia* Scheuer	AY789411	AY789412	AY789413
*Hymenoscyphus scutula* (Pers.) W. Phillips	AY789430	AY789431	AY789432
*Hypocrea lutea* (Tode) Petch	AF543768	AF543791	EU816393
*Lachnum bicolor* (Bull.) P. Karst.	AY544690	AY544674	U59005
*Lecanora concolor* Ramond	AY640993	AY640954	AF070037
*Leotia lubrica* (Scop.) Pers.	AY789358	AY789359	AY789360
*Leveillula taurica* (Lév.) G. Arnaud	AB033471	AB022387	AF073351
*Lophodermium pinastri* (Schrad.) Chevall	AF106014	AY004334	AF775701
*Loramyces juncicola* W. Weston	AF203464	UNPUBL.	UNPUBL.
*Meria laricis* Vuill.	AF106017	DQ470954	U92298
*Microglossum olivaceum* (Pers.) Gillet	AY789396	AY789397	AY789398
*Microglossum rufum* (Schwein.) Underw.	DQ257358	DQ257359	DQ257360
*Mitrula paludosa* Fr.	AY789422	AY789423	AY789424
*Mollisia cinerea* (Batsch) P. Karst.	DQ470990	DQ470942	DQ491498
*Monilinia fructicola* (G. Winter) Honey	AY544724	AY544683	DQ491506
*Neofabraea malicorticis* H.S. Jacks	AY544706	AY544662	AF281386
*Neolecta irregularis* (Peck) Korf & J.K. Rogers	AY789379	AY789380	AY789381
*Neurospora crassa* Shear & B.O. Dodge	AY046271	AF286411	AY046222
*Ochrolechia parella* (L.) A. Massal.	AF274109	AF274097	AF329174
*Orbilia auricolor* (A. Bloxam ex Berk.) Sacc.	AJ001986	AJ261148	U51952
*Orbilia delicatula* (P. Karst.) P. Karst.	U72603	AY261178	U72595
*Peltigera aphthosa* (L.) Willd.	AY424225	AF286759	AF158645
*Peziza phyllogena* Cooke	AY789327	AY789328	AY789329
*Peziza varia* (Hedw.) Fr.	AY789390	AY789391	AY789392
*Phacidiopycnis pyri* (Fuckel) Weindlm.	DQ470997	DQ470949	EU156058
*Phialocephala fortinii* C.J.K. Wang & H.E. Wilcox	AY524846	AF269219	AY347413
*Phyllactinia moricola* (Henn.) Homma	AB033481	AB022401	D84385
*Piceomphale bulgarioides* (Rabenh.) Svrcek	Z81388	Z81415	Z81441
*Pleochaeta shiraiana* (Henn.) Kimbr. & Korf	AB120750	AB022403	D84380
*Protomyces inouyei* Henn.	AY548295	EU056299	DQ497617
*Rhytisma* sp.	AF356695	AF356696	AY465516
*Rutstroemia firma* (Pers.) P. Karst.	DQ471010	DQ470963	U21824
*Saccharomyces cerevisiae* Meyen ex E.C. Hansen	J01353	J01355	AY247400
*Sarcoleotia* sp.	AY789298	AY789299	AY789300
*Sarea resinae* (Fr.) Kuntze	AY641004	AY640965	AY781237
*Schizosaccaromyces pombe* Lindner	Z19578	Z19578	Z19578
*Scleromitrula shiraiana* (Henn.) S. Imai	AY789406	AY789407	AY789408
*Sclerotinia sclerotiorum* (Lib.) de Bary	AY789346	AY789347	AF455526
*Sordaria fimicola* (Roberge ex Desm) Ces. & De Not.	AY545724	AY545728	EU918704
*Spathularia flavida* Pers.	AY789356	AF433142	AF433152
*Taphrina wiesneri* (Rathay) Mix	AY548293	AY548292	DQ497616
*Trapelia placodioides* Coppins & P. James	AF119500	AF274103	AF274081
*Trichoglossum hirsutum* (Pers.) Boud.	AY789312	AY789313	AY789314
*Uncinula septata* E.S. Salmon	AB183530	AB183532	AB183533
*Vibrissea flavovirens* (Pers.) Korf & J.R. Dixon	AY789425	AY789426	AY789427
*Vibrissea truncorum* (Alb. & Schwein.) Fr.	AY789401	AY789402	AY789403
*Xylaria hypoxylon* L. & Grev.	AY544692	AF132333	AF163035

Information about unpublished sequences is available from the AFTOL website (http://aftol.org).

### 2. Phylogenetic analyses

One data set was prepared based on sequences of 78 taxa from three nuclear genes, SSU rDNA, LSU rDNA and 5.8S rDNA. The LSU rDNA sequence of *Rutstroemia bolaris* was 527 bp shorter than in other taxa. No 5.8S rDNA data of *Hemiphacidium longisporum* were available. Sequences were aligned with ClustalX [21], then adjusted by eye in the data editor of PAUP* 4.0b [13]. Introns and short insertions were deleted, and ambiguously aligned positions were excluded from the data sets before performing the analyses. Alignments were deposited and are available at TreeBASE (accession number SN3978). A basidiomycete *Cotylidia* sp. was used to root the tree in the analyses. The data set was analyzed with PAUP* 4.0b and MrBayes 3.1.1 [14]. Gaps were treated as missing data.

Parsimony analyses were performed using equal weighting of characters and transformations. Heuristic searches were performed with one thousand replicate searches, each with a random taxon addition sequence, MAXTREES set to autoincrease, and TBR branch swapping. Robustness of individual branches was estimated by maximum parsimony bootstrap proportions (BP), using 500 replicates, each consisting of a heuristic search with 50 random taxon addition sequences, MAXTREES set to autoincrease, and TBR branch swapping. Bayesian posterior probabilities were computed using the Metropolis-coupled Markov chain Monte Carlo method (MCMCMC) under the GTR+Γ+I model in MrBayes 3.1.1 by running four chains with 2,000,000 generations. Trees were sampled every 100 generations. Likelihoods converged to a stable value after ca. 500,000 generations in the wider-range analyses and after ca. 100,000 generations in the narrower-range analysis, and all trees prior to the convergence were discarded as the “burn-in” phase before computing a consensus tree in PAUP*. Bayesian posterior probabilities (PP) were obtained from the 50% majority rule consensus of the remaining trees, and clades with PP≥0.95 were considered to be significantly supported.

### 2. Analysis of correlation between leaf-living ecology and reduced morphology

To evaluate the correlation between leaf-living ecology and reduced apothecium morphology across ascomycetes, BayesTraits [15] was used. All sampled 19000 trees from the above Bayesian analyses were used as the input treefile for the program [22]. An additional two-character data set for the 78 taxa was prepared as the input file. The characters and their binary character states were:

1. Whether or not a species is a leaf-endophyte. Many ascomycetes can have a endophytic stage in their life cycle, so here we used a strict sense of leaf-living that included only ascomycetes completing their whole life history in living leaves. The Rhytismatales is a large and diverse order, and most of its species are endophytes. Here we included three endophytic taxa to display the significant morphological change between the leaf endophytes and saprobic species in Cudoniaceae in this order. Members of the Erysiphales are leaf living fungi and share a similar ecology with leaf endophytes. Most Erysiphales fungi produce the somatic mycelia exclusively on leaf surfaces, with a kind of hyphal structure (a haustoria) penetrating the leaf surface and tissues for nutrition.
2. Whether or not a species features highly reduced ascomata morphology compared with their sister groups. Significant reductions of the ascomata have occurred multiple times across ascomycetes. Ascomycetes only known by their asexual stage or yeasts, which are not leaf-endophytes, were considered as highly reduced taxa in our analyses.

Using these tree and input files, an independent and a dependent model for discrete traits evolution were selected in BayesTraits. Several MCMC runs were performed on both models with 2000,000 iterations. The MCMC chain was sampled every 100 iterations after a burn-in of 1000 iterations. To achieve an acceptance rate of between 20 and 40%, a ratedev value of 2 or 8 was chosen for independent or dependent model individually. A hyperprior procedure was used to establish the parameter for an exponential prior. A reversible jump was applied, seeding the exponential prior from a uniform on the interval 0 to 30.
